# Presence but not number of secondary type mutations influences outcome in de novo AML without MDS‐associated or recurring cytogenetic abnormalities

**DOI:** 10.1002/jha2.710

**Published:** 2023-05-18

**Authors:** Olga K. Weinberg, Miguel D. Cantu, Jeffrey Gagan, Yazan F. Madanat, Daniel A. Arber, Robert P. Hasserjian

**Affiliations:** ^1^ Department of Pathology University of Texas Southwestern Medical Center Dallas Texas USA; ^2^ Department of Internal Medicine University of Texas Southwestern Medical Center Dallas Texas USA; ^3^ Harold C. Simmons Comprehensive Cancer Center University of Texas Southwestern Medical Center Dallas Texas USA; ^4^ Department of Pathology University of Chicago Chicago Illinois USA; ^5^ Department of Pathology Massachusetts General Hospital Boston Massachusetts USA

**Keywords:** AML, GENETICS, MUTATIONS

## Abstract

A group of gene mutations has been identified to be strongly associated with secondary acute myeloid leukemias (AML) arising from prior myeloid neoplasms. The International Consensus Classification (ICC) and proposed 5th edition of the World Health Organization (WHO) classification differ by inclusion of *RUNX1*. A recent study suggested that having two or more secondary mutations is associated with a particularly poor prognosis. In a study of 294 de novo AML patients, we found that patients with at least one ICC‐defined secondary mutation had shorter survival when compared to those without secondary mutations, and ICC/WHO groups of two or more mutations did not predict for worse outcomes.

## 1 INTRODUCTION

Acute myeloid leukemias (AML) are hematological neoplasms typically associated with rapid onset and often chemo‐resistant disease. In the proposed 5th edition of the World Health Organization (WHO) classification (WHO5), patients with AML and a known history of myelodysplastic neoplasm (MDS) or MDS/myeloproliferative neoplasms (MPN) are classified together with those patients with AML and MDS‐related cytogenetic abnormalities and/or gene mutation [1], while in the 2022 International Consensus Classification (ICC), a history of MDS or MDS/MPN is now denoted as a diagnostic qualifier, and MDS‐related cytogenetics and MDS‐related gene mutations denote distinct AML subgroups [2]. In both the WHO5 and ICC, the finding of morphologic dysplasia in AML is no longer used to distinguish de novo and secondary AML. A group of gene mutations has been identified to be strongly associated with secondary AML arising from prior myeloid neoplasms [2, 3, 4, 5, 6]. Lindsley et al. found that the presence of a mutation in *SRSF2*, *SF3B1*, *U2AF1*, *ZRSR2*, *ASXL1*, *EZH2*, *BCOR*, or *STAG2* was >95% specific for the diagnosis of secondary AML [5]. Papaemmanuil et al. confirmed lower survival rates and higher relapse rates in patients with chromatin‐spliceosome mutations, which were defined by mutations in genes regulating RNA splicing (*SRSF2*, *SF3B1*, *U2AF1*, and *ZRSR2*), chromatin (*ASXL1*, *STAG2*, *BCOR*, *MLLPTD*, *EZH2*, and *PHF6*), or transcription (*RUNX1*) [3]. WHO5 includes a mutation‐based definition of AML, myelodysplasia‐related (AML‐MR) group, based on a set of eight genes—*SRSF2*, *SF3B1*, *U2AF1*, *ZRSR2*, *ASXL1*, *EZH2*, *BCOR*, and *STAG2* [1]. ICC includes mutations in any of those eight genes and/or *RUNX1* mutation to define a diagnosis of AML‐MR gene mutations (which incorporates the prior entity of AML with mutated *RUNX1*) [2]. A recent study by Tazi et al. suggested that having two or more secondary mutations is associated with a particularly poor prognosis versus the requirement of only a single mutation for MDS‐related AML [7]. This study included *NF1*, *CUX1*, *PHF6*, *RUNX1*, *SETBP1*, and *MLL* ^PTD^ mutations, in addition to the group of eight mutations (*SRSF2*, *SF3B1*, *U2AF1*, *ZRSR2*, *ASXL1*, *EZH2*, *BCOR*, or *STAG2*), in the secondary mutation group. The Tazi et al. secondary mutation group presented with lower blast counts and higher incidence of antecedent hematologic disease, but significant association with adverse outcomes was limited to patients with two or more mutations [7].

The majority of AML cases present de novo, without any known history of an antecedent myeloid neoplasm or exposure to cytotoxic therapy and are associated with heterogenous clinical outcomes, necessitating risk stratification at the time of diagnosis by clinicopathologic and genetic criteria [8]. We sought to determine the significance of 1 versus ≥2 secondary‐type mutations in a series of de novo AML cases lacking specific recurrent cytogenetic abnormalities in order to evaluate the impact of ICC and WHO‐defined secondary mutations on patient outcomes.

## 2 METHODS

We retrospectively identified de novo AML cases lacking cytogenetic features of AML with recurrent genetic abnormalities (AML‐RGA) or AML with myelodysplasia‐related changes (AML‐MRC) per revised 4th edition WHO classification; cases with history of cytotoxic therapy or any prior myeloid neoplasm were excluded [9]. Patient and clinical characteristics, including treatments administered, patient follow‐up, and outcome measures, were collected using the electronic health record. This study was approved by the institutional review boards of all participating institutions.

Next‐generation sequencing (NGS) was performed on specimens taken either at the time of initial diagnosis or after the initial diagnosis in patients who had not been treated by any disease‐modifying therapies. NGS panels included assessment of all eight WHO secondary mutations, all nine ICC secondary mutations, and all 14 secondary mutations in the Tazi et al. study, with the exception of *KMT2A*‐PTD, which was not assessed. Cases were classified based on whether they had 0, 1, or ≥2 secondary‐type (SM0, SM1, SM2) mutations according to the Tazi grouping, ICC grouping, or WHO grouping. Chi‐squared or Fisher's exact tests, as appropriate, were used to assess correlations between categorical variables. The Wilcoxon rank‐sum test was used to assess differences in continuous variables between groups. Overall survival (OS) and event‐free survival (EFS) were assessed using Kaplan–Meier method and groups were compared using log rank test.

## 3 RESULTS

A total of 294 patients of de novo AML were included in the study, with a median age of 65 years (range 18–100). Sixty‐one patients (21%) had a non‐MDS‐related abnormal karyotype, with most common abnormalities including trisomy 13 (7, 11%), trisomy 8 (15, 24%), and trisomy 11 (8, 13%); there was no difference in rate of abnormal karyotype between SM0, SM1, and SM2 patients (all *p* > 0.5).

Using Tazi et al. classification, there were 106 (36%) SM2 patients and 70 (24%) SM1 patients, while the remaining patients lacked secondary‐type mutations (SM0). Using ICC definition, there were 92 (31%) SM2 patients and 80 (27%) SM1 patients, and using the WHO definition, there were 68 (23%) SM2 patients, 86 (29%) SM1 patients, and the remaining patients were SM0. *RUNX1* mutation was present in 72 patients but co‐occurred with other secondary mutations in 54 patients (18%). Tazi‐SM2 patients presented with lower white blood cells (*p* = 0.012) and lower blasts in peripheral blood (*p* = 0.014) and bone marrow (*p* = 0.015) as compared to SM0 patients (Table 1). Similar findings were seen in ICC‐SM2 and WHO‐SM2 patients as compared to SM0 (*p* < 0.05). Tazi‐SM2 patients were significantly older than Tazi‐SM0 patients (*p* = 0.01); however, only trend toward older age was seen compared to Tazi‐SM1 patients (*p* = 0.06). Similar findings were seen in ICC‐SM2 and WHO‐SM2 patients. There was no correlation between age, total number of mutations, presence of cytogenetic abnormalities, or mutations specifically in *RUNX1*, *ASXL1*, *NF1*, or *SRSF2* genes with outcome as measured by OS or EFS in this cohort (all *p* > 0.1).

**TABLE 1:** Clinical presentation of acute myeloid leukemia (AML) patients with Tazi‐defined secondary mutations.

	AML ≥2 secondary mutations (*N* = 106)	AML with 1 secondary mutation (*N* = 70)	*p*‐Values (≥2 vs. 1 mutation)	AML with no secondary mutations (*N* = 114)	*p*‐Values (≥2 vs. no mutations)	*p*‐Values (1 vs. 0 mutations)
Age	68	64	0.06	59	0.01	0.01
WBC (×10^9^/L, median)	3.6	10.7	0.03	19.5	<0.0001	NS
PB blast (%, median)	5	14.5	0.07	33.5	<0.0001	0.04
Hgb (×10^9^/L, median)	8.8	8.4	NS	9.1	NS	NS
Platelets (×10^9^/L, median)	63	71	NS	70	NS	NS
BM blast (%, median)	52	60	NS	69	0.001	0.02

Abbreviations: BM, bone marrow; Hgb, hemoglobin; PB, peripheral blood; WBC, white blood cells.

In patients treated with induction or hypomethylating agents (*n* = 282), Tazi‐SM1 patients showed shorter OS (*p* = 0.008) but not EFS (*p* = 0.13) when compared to Tazi‐SM0. Tazi‐SM2 showed worse outcomes as compared Tazi‐SM0 (OS, *p* = 0.0049; EFS, *p* = 0.0018), but no difference was seen when compared to Tazi‐SM1 (OS, *p* = 0.38; EFS, *p* = 0.62). Among patients treated with induction (*n* = 223), Tazi‐SM1 patients showed shorter OS (*p* = 0.006) and EFS (*p* = 0.024) when compared to Tazi‐SM0. Tazi‐SM2 exhibited worse outcome compared to Tazi‐SM0 (OS, *p* = 0.0033; EFS, *p* = 0.0018), but no difference was seen when compared to Tazi‐SM1 (OS, *p* = 0.42; EFS, *p* = 0.56).

**FIGURE 1:**
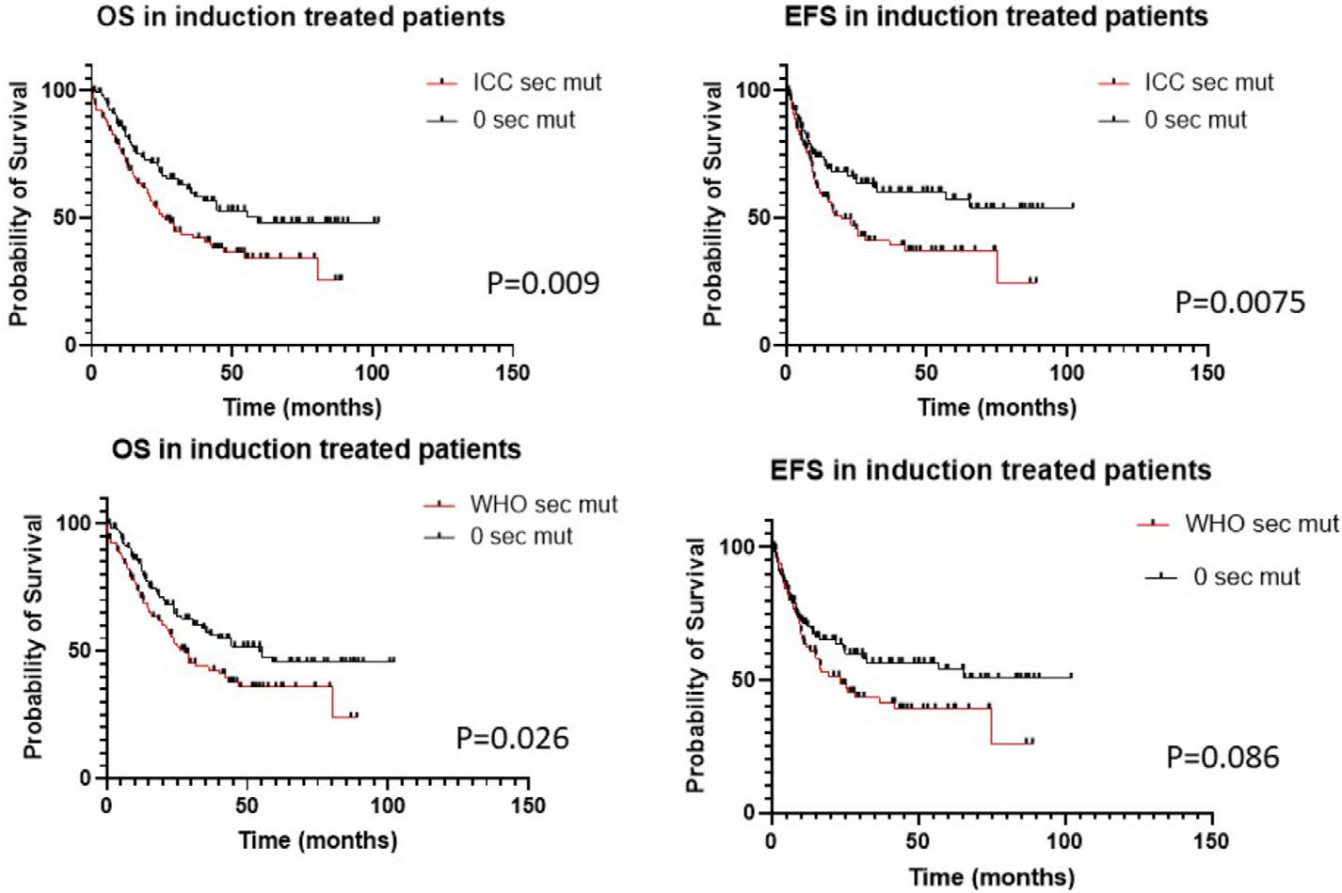
Comparison of patients with International Consensus Classification (ICC) and World Health Organization (WHO) defined secondary mutations.

In patients treated with induction and Hypomethylating agents (HMA), ICC‐SM1 group showed significantly shorter OS and trend toward shorter EFS (*p* = 0.007 and 0.06) as compared with ICC‐SM0. WHO‐SM1 showed only shorter OS but for EFS (OS, *p* = 0.02; EFS, *p* = 0.33) as compared with WHO‐SM0. In patients who received induction chemotherapy (Figure 1), ICC‐SM1 group demonstrated significantly shorter OS and EFS (*p* = 0.009 and 0.0075) as compared with ICC‐SM0. WHO‐SM1 group showed only shorter OS (*p* = 0.03) but not EFS (*p* = 0.09) as compared to WHO‐SM0. (Figure 1). When comparing ICC‐SM2 with ICC‐SM1 or ICC‐SM0, there was no significant difference in OS and EFS (*p* > 0.05). Similar findings were seen in comparison of WHO‐SM2 with WHO‐SM1 and WHO‐SM0.

In multivariable analysis (Table S1), presence of ICC‐SM1 (*p* = 0.038) and Tazi‐SM2 (*p* = 0.004) predicted for shorter OS, while WHO‐SM1 was not significant. Similar findings were also seen in multivariable analysis for EFS (Table S2).

## 4 DISCUSSION

The AML‐MRC group was an attempt to identify a subgroup of AML biologically related to MDS, with older age, lower blast counts, lower remission rates, multilineage dysplasia, and shorter OS. Various gene mutations have been reported to be commonly associated with AML arising from MDS and in the background of therapy‐related disease and have been proposed alongside MDS‐associated cytogenetic abnormalities as a further defining feature of AML‐MRC. One of the goals of the recently developed ICC and proposed 5th edition of the WHO classification of acute leukemias was to move to a genetically defined classification, and thus, both classifications now recognize MDS‐associated mutations as defining myelodysplasia‐related AML, with only difference being inclusion of *RUNX1* in ICC. *RUNX1*‐mutated AML has historically been associated with resistance to conventional cytotoxic chemotherapy and with significantly lower complete remission rates and inferior OS [10, 11]. Other studies have suggested that the prognosis of mutated *RUNX1* AML is context dependent, with worse outcomes observed mainly in patients with *ASXL1*, *SRSF2*, and/or *PHF6* co‐mutations [12]. We found that ICC‐SM1 group was associated with shorter OS and EFS regardless of therapy when compared with ICC‐SM0, unlike WHO‐SM1 group, which was only associated with worse OS.

Tazi et al. found that two or more secondary mutations are associated with a particularly poor prognosis versus the requirement of only a single mutation for MDS‐related AML and included additional mutations in *RUNX1*, as well as *NF1*, *CUX1*, *PHF6*, *SETBP1*, and *KMT2A^PTD^* in defining secondary mutations [7]. *PHF6* and *NF1* mutations have been reported to be associated with worse prognosis in AML, including intermediate‐risk subtype [13, 14], while *CUX1* mutations have been reported to be more common in secondary AML [15]. We confirm that Tazi‐SM2 patients show worse outcomes, but only when compared to Tazi‐SM0. In our study, WHO‐SM2 and ICC‐SM2 were not associated with adverse prognosis, and the difference may be due to inclusion of these different combinations of mutations in Tazi et al. study. The difference is unlikely related to cytogenetic abnormalities since Tazi et al. excluded complex karyotype [7].

We found that in the setting of de novo disease, AML patients with at least one secondary mutation had shorter OS and EFS, validating their inclusion as an adverse risk factor in the 2022 European Leukemia Net guidelines and as defining feature of MDS‐related AML in the 2022 ICC and WHO classifications. Our data also suggest that inclusion of *RUNX1* is appropriate in the group of MDS‐related gene mutations defined by the ICC, based on its impact on prognosis (Table 2).

**TABLE 2:** Most frequent secondary mutations (sec mut) listed in each group.

	ICC sec mut (%), *N* = 172	WHO sec mut (%), *N* = 154	Tazi sec mut (%), *N* = 106	Tazi >2 sec mut (%), *N* = 176
*RUNX1* ^a^	42	37	40	56
*SRSF2*	37	40	36	47
*ASXL1*	33	36	32	41
*BCOR*	20	22	19	24
*STAG2*	18	19	17	22
*U2AF1*	12	13	11	16
*SF3B1*	9	10	8	8
*ZRSR2*	2	3	2	2
*EZH2*	5	6	5	6
*NF1* ^b^	34	32	35	41
*SETBP1* ^b^	4	4	4	6
*PHF6* ^b^	3	3	4	5

^a^ Considered secondary‐type mutation in International Consensus Classification (ICC) but not World Health Organization (WHO).

^b^ Included in Nat Commun. 2022;13(1):4622 but not ICC or WHO classification.

## AUTHOR CONTRIBUTIONS

Olga K. Weinberg, Robert P. Hasserjian, Daniel A. Arber, and Miguel D. Cantu designed the study and collected data. Olga K. Weinberg analyzed the data and wrote a draft of the manuscript. Olga K. Weinberg, Robert P. Hasserjian, Daniel A. Arber, Miguel D. Cantu, Yazan F. Madanat, and Jeffrey Gagan extensively edited the manuscript.

## CONFLICT OF INTEREST STATEMENT

The authors declare no conflicts of interest.

## FUNDING INFORMATION

The authors received no specific funding for this study.

## ETHICS STATEMENT

The authors have confirmed ethical approval statement is not needed for this submission.

## PATIENT CONSENT STATEMENT

The authors have confirmed patient consent statement is not needed for this submission.

## PERMISSION TO REPRODUCE MATERIAL FROM OTHER SOURCES

None needed.

## CLINICAL TRIAL REGISTRATION

The authors have confirmed clinical trial registration is not needed for this submission.

## Supporting information

Supporting Information

## Data Availability

The data that support the findings of this study are available from the corresponding author upon reasonable request.
