# Supplementary material for: Presence but not number of secondary type mutations influences outcome in de novo AML without MDS‐associated or recurring cytogenetic abnormalities
Source: EJHaem. 2023 May 18;4(3):760–4. doi: 10.1002/jha2.710 (PMC10435713; doi:10.1002/jha2.710)
Supplement: Supplementary file 1 — Supporting Information [file JHA2-4-760-s001.docx]

**Supplementary Table 1**

Overall survival multivariable analysis including ICC-defined secondary mutations (ICC-SM1), WHO-defined secondary mutations (WHO-SM1) and Tazi-defined two secondary mutations (Tazi-SM2) and one secondary mutation (Tazi-SM1)

| Variable | Value | Standard error | Wald Chi-Square | Pr > Chi² | Hazard ratio | Hazard ratio Lower bound (95%) | Hazard ratio Upper bound (95%) |
| --- | --- | --- | --- | --- | --- | --- | --- |
| *FLT3-ITD* | 0.576 | 0.206 | 7.835 | 0.005 | 1.779 | 1.189 | 2.663 |
| Therapy | -0.437 | 0.224 | 3.828 | 0.050 | 0.646 | 0.417 | 1.001 |
| ICC-SM1 | 0.395 | 0.182 | 4.729 | 0.030 | 1.484 | 1.040 | 2.119 |
| BMT | -0.875 | 0.197 | 19.790 | <0.0001 | 0.417 | 0.000 | 0.613 |

| Variable | Value | Standard error | Wald Chi-Square | Pr > Chi² | Hazard ratio | Hazard ratio Lower bound (95%) | Hazard ratio Upper bound (95%) |
| --- | --- | --- | --- | --- | --- | --- | --- |
| *FLT3-ITD* | 0.558 | 0.207 | 7.282 | 0.007 | 1.747 | 1.165 | 2.621 |
| Therapy | -0.455 | 0.226 | 4.039 | 0.044 | 0.634 | 0.000 | 0.989 |
| WHO-SM1 | 0.288 | 0.180 | 2.548 | 0.110 | 1.333 | 0.937 | 1.898 |
| BMT | -0.868 | 0.197 | 19.456 | <0.0001 | 0.420 | 0.000 | 0.617 |

| Variable | Value | Standard error | Wald Chi-Square | Pr > Chi² | Hazard ratio | Hazard ratio Lower bound (95%) | Hazard ratio Upper bound (95%) |
| --- | --- | --- | --- | --- | --- | --- | --- |
| *FLT3-ITD* | 0.653 | 0.211 | 9.569 | 0.002 | 1.922 | 1.270 | 2.907 |
| Therapy | -0.446 | 0.223 | 4.004 | 0.045 | 0.640 | 0.000 | 0.991 |
| Tazi-SM1 | 0.197 | 0.221 | 0.791 | 0.374 | 1.217 | 0.789 | 1.878 |
| Tazi-SM2 | 0.592 | 0.206 | 8.221 | 0.004 | 1.807 | 1.206 | 2.707 |
| BMT | -0.889 | 0.198 | 20.274 | <0.0001 | 0.411 | 0.000 | 0.605 |

**Supplementary Table 2**

Event free survival multivariable analysis including ICC-defined secondary mutations (ICC-SM1), WHO-defined secondary mutations (WHO-SM1) and Tazi-defined two secondary mutations (Tazi-SM2) and one secondary mutation (Tazi-SM1)

| Variable | Value | Standard error | Wald Chi-Square | Pr > Chi² | Hazard ratio | Hazard ratio Lower bound (95%) | Hazard ratio Upper bound (95%) |
| --- | --- | --- | --- | --- | --- | --- | --- |
| *FLT3-ITD* | 0.466 | 0.230 | 4.092 | 0.043 | 1.593 | 1.015 | 2.502 |
| Therapy | 0.170 | 0.269 | 0.402 | 0.526 | 1.186 | 0.700 | 2.009 |
| ICC-SM1 | 0.415 | 0.200 | 4.324 | 0.038 | 1.515 | 1.024 | 2.241 |
| BMT | -0.731 | 0.213 | 11.724 | 0.001 | 0.482 | 0.000 | 0.732 |

| Variable | Value | Standard error | Wald Chi-Square | Pr > Chi² | Hazard ratio | Hazard ratio Lower bound (95%) | Hazard ratio Upper bound (95%) |
| --- | --- | --- | --- | --- | --- | --- | --- |
| *FLT3-ITD* | 0.418 | 0.230 | 3.318 | 0.069 | 1.519 | 0.969 | 2.383 |
| Therapy | 0.115 | 0.271 | 0.179 | 0.672 | 1.122 | 0.659 | 1.910 |
| WHO-SM1 | 0.227 | 0.197 | 1.333 | 0.248 | 1.255 | 0.853 | 1.847 |
| BMT | -0.712 | 0.213 | 11.193 | 0.001 | 0.491 | 0.000 | 0.745 |

| Variable | Value | Standard error | Wald Chi-Square | Pr > Chi² | Hazard ratio | Hazard ratio Lower bound (95%) | Hazard ratio Upper bound (95%) |
| --- | --- | --- | --- | --- | --- | --- | --- |
| FLT3-ITD | 0.490 | 0.233 | 4.402 | 0.036 | 1.632 | 1.033 | 2.578 |
| Therapy | 0.157 | 0.268 | 0.341 | 0.559 | 1.169 | 0.692 | 1.978 |
| Tazi-SM2 | 0.323 | 0.239 | 1.822 | 0.177 | 1.381 | 0.864 | 2.208 |
| Tazi-SM1 | 0.472 | 0.229 | 4.265 | 0.039 | 1.604 | 1.024 | 2.511 |
| BMT | -0.734 | 0.214 | 11.787 | 0.001 | 0.480 | 0.000 | 0.730 |
